# Optimizing culturing conditions in patient derived 3D primary slice cultures of head and neck cancer

**DOI:** 10.3389/fonc.2023.1145817

**Published:** 2023-03-30

**Authors:** Maria do Carmo Greier, Annette Runge, Jozsef Dudas, Lukas Carpentari, Volker Hans Schartinger, Avneet Randhawa, Melissa Mayr, Monika Petersson, Herbert Riechelmann

**Affiliations:** ^1^ Department of Otorhinolaryngology, Head and Neck Surgery, Medical University of Innsbruck, Innsbruck, Austria; ^2^ Department of Otolaryngology, Head and Neck Surgery, Rutgers New Jersey Medical School, Newark, NJ, United States; ^3^ ViraTherapeutics GmbH, Rum, Austria

**Keywords:** head and neck cancer, tumor microenvironment, cultured neoplastic cells, platelet rich fibrin, culture media

## Abstract

**Background:**

Three-dimensional primary slice cultures (SC) of head and neck squamous cell carcinomas (HNC) are realistic preclinical models. Until now, preserving structure and viability *ex vivo* for several days has been difficult. The aim of this study was to optimize cultivation conditions for HNC SC and analyze the added effects of platelet rich fibrin (PRF) on these conditions.

**Methods:**

SC were prepared from the tumor biopsies of 9 HNC patients. Cultures were incubated for 1 and 7 days in three different media- Keratinocyte serum-free medium (SFM), RPMI-1640i, and 1:1 mix of both, with and without addition of PRF. After culturing, SC were fixated, embedded, and stained with Hematoxylin-Eosin (HE) and cleaved caspase-3. In addition, triple immune fluorescence staining for cytokeratin, vimentin and CD45 was performed. Outcome parameters were cell count and cell density, viability and apoptosis, SC total area and proportions of keratinocytes, mesenchymal and immune cells. The effects of culture time, medium, and addition of PRF were calculated in an SPSS generalized linear model and using the Wald Chi-Squared test.

**Results:**

Ninety-four slice cultures were analyzed. Viability remained stable for 7 days in culture. After addition of PRF, cell viability increased (p=0.05). SC total area decreased (0.44 ± 0.04 mm^2^ on day 1 (95% CI: 0.35 to 0.56) to 0.29 ± 0.03 mm^2^ on day 7 (95% CI: 0.22 to 0.36), but cell density and cell proportions remained stable. Differences in cultivation media had no significant impact on outcome parameters.

**Conclusion:**

HNC SC can be preserved for up to 7 days using the tested cultivation media. Cell viability was best preserved with addition of PRF. HNC SC are a versatile experimental tool to study physiology and drug actions. Autologous PRF can help simulate realistic conditions *in vitro*.

## 1 Introduction

Head and neck squamous cell carcinomas (HNC) are heterogeneous tumors with highly variable cellular composition, invasion patterns, and therapy response (1). Realistic preclinical models for personalized therapy strategies are lacking. Cell cultures are currently the standard models (2) of basic tumor mechanisms (3). However, without the tumor microenvironment (TME), cell cultures are limited in their translational applicability (4, 5). Tumor architecture, physiological state, and interactions among different cell types (6) can play important roles in cancer progression and invasion (7, 8). Recently, patient-derived HNC models with the TME have been used to study tumor-specific characteristics and develop individualized therapies (8–11). Such TME models can be *in-vivo*, two- and three-dimensional co-cultures (11, 12), patient derived xenograft models (13, 14), organoids (14, 15), microfluidic designs (16–18), organ-on-a-chip models (19, 20), spheroids (21), 3D bio prints (22, 23), 3D collagen-based scaffolds (24) and advanced three-dimensional spheroid models from dissected whole tumor tissues. However, in all of the above-mentioned models, the *in situ* spatial arrangement of the TME is dissolved and interactions with neighboring tissue may be altered (24–26). Primary slice cultures (SC) are 250 to 400 μm thick slices of tumor tissue samples in which the original 3D structures and organization of the TMEs are preserved (4). As SC are open systems, substances can easily be added and therapeutic efficacy and resistance mechanisms may be directly studied (27). The impact of the HNC cell- microenvironment interaction on invasion patterns and response to antitumoral treatment has become more apparent during the last few years. Different 3D preclinical models of HNC have thus been established recently to study tumor cell characteristics in a “close to real life environment” over the course of several days in a laboratory setting. 3D cultures of oropharyngeal squamous cell carcinoma cell lines were mounted on a collagen – based scaffold to study expression of markers of epithelial- mesenchymal transition as well as matrix interactions and migration behavior and drug resistance pathways both *in vitro* and as xenografts (28). Gerlach and coauthors first described cultivation of HNC SC and observed the effect of cytotoxic drugs for up to 7 days (29). Three-dimensional organotypic co-culture models mounted on a dermal equivalent of fibroblasts and viscose fibers were successfully cultured for 7- 21 days to study proliferation, infiltrative growth patterns and distribution of cancer associated fibroblasts and leukocytes depending on HPV status (30). Furthermore, the applicability of HNC 3D SC as a platform to study novel therapeutic approaches such an oncolytic virus was recently described by Runge and Mayr et al. (31). However, viability and cell proportions may vary due to heterogeneity of HNC tumor tissue samples, effects of culture media, and SC incubation times. Furthermore, *ex vivo* viability of HNC SC might be compromised due to a lack of several autologous growth factors. Platelet rich fibrin (PRF), a completely autologous substance obtained by centrifugation of peripheral venous blood, contains platelets, leukocytes and several biologically active proteins including platelet alpha granules, platelet−derived growth factor (PGDF), transforming growth factors−β (TGF−β), vascular endothelial growth factors (VEGF), and epidermal growth factors (32, 33) in a fibrin matrix. PRF has been suggested to have beneficial effects on viability of tumor explants (32, 34).

The main objective of this study was to optimize HNC SC culturing conditions over a period of seven days. Quality of SC of different media were compared after 1 and 7 days. The effects of autologous PRF on cell viability in HNC SC and selective effects of different culturing media and PRF on epithelial cells, fibroblasts and immune cells were studied.

## 2 Methods

### 2.1 Study design

In this study in HNC SC, effects of 3 experimental parameters were examined: cultivation time of 1 day and 7 days, three cultivation media (Keratinocyte SFM, RPMI1640, 1:1 mix of both), and addition of autologous patient derived PRF. The outcome parameters included SC total area (mm2), the total number of cells in relation to the area (the “cell density”, cells/mm2), viability (%), number of tumor cells, fibroblasts, and leukocytes per mm2, and their relative proportions.

### 2.2 Patients

Tumor tissue samples from nine patients with suspected incident, locally advanced head and neck squamous cell carcinomas were collected, who underwent endoscopy under anesthesia as part of their initial staging between October 2020 and June 2021 at the Department of Otorhinolaryngology, Head and Neck Surgery, Medical University of Innsbruck (**Table 1**). The samples were always collected from the primary tumor. Approval for this study was obtained from the Ethics Committee of the Medical University of Innsbruck (EK-number: 1199/2019, date of approval: 19/05/2019). Written informed consent was obtained from all patients. Inclusion criteria comprised of patient age over 18 years, endoscopy under anesthesia, and locally advanced primary tumor (T3-T4). Patients were excluded if there was a contraindication for endoscopy under anesthesia or if they had received prior treatment for HNC.

**Table 1:** Patient characteristics and tumor locations.

Patient	Sex	Age	Location of primary tumor	TNM	p16 status
1	male	61	Oropharynx	cT4 cN1 cM0	positive
2	female	52	Oropharynx	cT4a cN2c cM1	negative
3	male	73	Larynx	cT4a cN1 cM0	negative
4	male	72	Larynx	cT3 cN2c cM0	negative
5	male	54	Oropharynx	cT3 cN2c cM0	negative
6	male	64	Hypopharynx	cT4a cN3b cM0	negative
7	male	59	Hypopharynx	cT3 cN3b cM0	negative
8	female	87	Oropharynx	cT3 cN1 cM0	negative
9	female	31	Nasopharynx	cT1 cN0 cM0	negative

### 2.3 Explant collection and cutting

**Figure 1:**
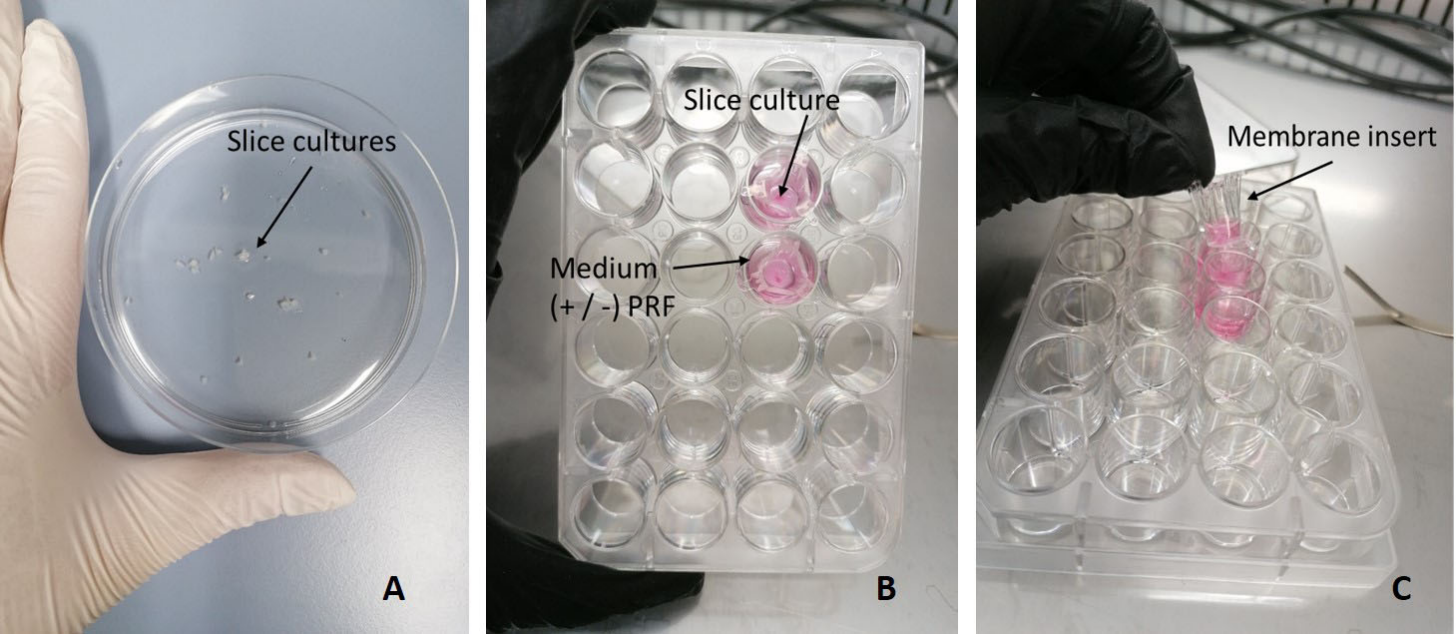
Culture setup of SC. **(A)** SC after being cut with the Vibratome. **(B)** SC cultivated in a 24-well plate, submerged in medium with or without PRF. **(C)** Hanging membrane insert.

Samples with a diameter of > 4 mm³ were collected with biopsy forceps from a non-necrotic tumor area during endoscopy under anesthesia (13). After submersion in Medium 199 (#31150022, Thermo Fischer Scientific, Rochester, NY, USA), the samples were immediately transferred to the laboratory for installation of SC. Afterwards, 12 slices with a thickness of 300 μm were cut from each sample with the vibratome (VT1200S Leica, Wetzlar, Germany; **Figure 1A**).

### 2.4 Autologous platelet rich fibrin

To prepare PRF, one S-PRF tube (#S-PRF, Mectron, Köln, Germany) of 4.9 ml of peripheral venous blood was obtained by cubital venipuncture from the same HNC patients. The blood sample was centrifuged immediately (Choukroun - PRF Duo Quattro System centrifuge, Cologne, Germany) at 44g for 8 min without addition of any substances. After centrifugation, 50µl of PRF were taken from the tube and added to the corresponding well with a syringe.

### 2.5 Cultivation media

For optimization of culturing conditions, separate examination of HNC SC in three different media with and without serum, growth factors, and PRF was performed (35). SC were submerged in hanging membrane inserts of a 24-well plate (Corning Incorporated-Life sciences, Durham, USA; **Figures 1B, C**). 50 μL of patient derived autologous PRF were added to 12/24 inserts. Afterwards, 150 μL of the respective medium were added. The media used were Keratinocyte SFM enriched with human recombinant epidermal growth factor (rEGF), bovine pituitary extract (BPE) (serum free medium; #10724-011, Gibco, Grand Island, NY, USA), RPMI-1640 (#31150022 Thermo Fisher Scientific, Rochester, NY, USA) with additional 10% fetal bovine serum (FBS), and a 1:1 mix of Keratinocyte SFM and RPMI-1640 in a 50 ml Falcon tube (#10788561, Szabo Scandic, Vienna, Austria). All media were supplemented with Gibco Antibiotic-Antimitotic (100x) (#15240062, Thermo Fischer Scientific, Rochester, NY, USA), a mix of three antibiotics (10.000 µg/mL streptomycin, 25 µg/mL amphotericin B and 10.000 units/mL penicillin). SC were then incubated in a 37°C incubator (5% CO2) over 1 and 7 days.

### 2.6 Fixation and embedding

After cultivation periods of 1 and 7 days, SC were fixed in 4% paraformaldehyde (#FN-10000-4-1, SAV Liquid Production GMBH, Flintsbach am Inn, Germany) overnight (4° C) and washed with phosphate-buffered saline (PBS) (Fresenius Kabi GmbH, Bad Homburg vor der Höhe, Germany) the next day. Fixed SC were embedded in HistoGel (#HG-4000-012, Thermo Scientific, Massachusetts, USA) and prepared for paraffin embedding *via* dehydration and paraffin impregnation with the Histos 5 microwave system (Milestone, Bergamo, Italy). All steps were performed as described by Lechner M et al. (36). Afterwards, hardened paraffin blocks were cut into 5µm slices with the HM 355S microtome (Microm, Walldorf, Germany) and transferred onto Superfrost Plus slides (Menzel, Braunschweig, Germany).

### 2.7 Staining procedures

Prior to staining, slides were dewaxed following the protocol of Giotakis et al. (37). Hematoxylin-Eosin (HE) staining was done following the manufacturers´ protocol (#1.05174.0500, Merck KGaA, Darmstadt, Germany). Following the protocol of Fischer N et al., cleaved caspase-3 (CC3) staining was performed using the fully automated immunostaining system (Ventana Roche Discovery Classic, Tucson, AZ, USA) and the CC3 antibody (1:400x, polyclonal rabbit, #9661, Cell Signaling Technology, MA, USA) (36). Epithelial, mesenchymal and immune cells were analyzed with triple immunofluorescence staining, containing conjugated vimentin (eFluor 570 conjugated) (#14-9897-82, eBioscience™, ThermoFischer, Waltham, MA United States), cytokeratin (CK) (Alexa Fluor 488 conjugated) (#628601, BioLegend, San Diego, United States), and CD45 (Alex Fluor 594 conjugated (#304060, BioLegend, San Diego, United States) as described by Giotakis et al. (37). Per slide, 8 μL of CD45 and 4 μL CK and vimentin, respectively, were used and mixed with 100 μL of antibody diluent (Roche, Ventana, Tucson, AZ, USA). The mix was then pipetted manually on the slides in the Ventana Discovery Slide Autoimmunostainer. For nuclear counterstain, 4′,6-diamidin-2-phenylindolethen (DAPI, 1:46.000, Thermo Fisher Scientific, Darmstadt, Germany) was pipetted onto the slides manually. To reduce auto fluorescence, the Vector TrueVIEW Auto Fluorescence Quenching Kit (#VEC-SP-8400, Vector Laboratories, Burlingame, California, USA) was used (37). After staining procedures, the slides were mounted with Vectashield Vibrance (Vector Laboratories) and dried overnight (38).

### 2.8 SC quality rating

Gross quality of SC was rated microscopically after 1 and 7 days of cultivation on HE staining. Texture and compactness of the tissue, viability, and necrotic areas were rated from 0 to 3, with 0 as the lowest and 3 as the highest quality score (**Table 2**).

**Table 2:** Scoring system (0-3) for SC quality in HE stained slices.

Score	Description
**0**	≤20% viable cells
**1**	21-40% viable cells
**2**	41-60% viable cells
**3**	≥60% viable cells

### 2.9 Image acquisition and analysis

TissueFAXS (TissueGnostics GmbH, Vienna, Austria) was used for image acquisition. Viability score, SC total area, total cell count, and total count of CC3 positive and-negative cells were analyzed in HE and CC3 stainings with HistoQuest according to Ingruber et al. (37) and Steinbichler et al. (39). In IF stained images, proportions and distributions of epithelial tumor cells, fibroblasts, and leukocytes were analyzed with TissueQuest according to Giotakis et al. (35). Tumor cells were defined in fluorescence-stained slices as cytokeratin or combined cytokeratin-vimentin-stained cells. Fibroblasts were defined as single vimentin-stained cells and leucocytes as single CD45 or CD45- vimentin combined stained cells. 

### 2.10 Data analysis

Given the right-skewed distributions of the outcome parameters, a generalized linear model was used, assuming a gamma distribution and a logistic link function. All main effects and interactions were included. Parameters were estimated using the maximum likelihood method, and p values and confidence intervals were calculated according to Wald. Calculations were performed with SPPS Statistics Ver. 27 (IBM, Armonk, NY). Estimated marginal means (EMM) and their standard errors (SEM) were graphically presented using GraphPad Prism 9 (GraphPad Software, San Diego, CA, USA).

## 3 Results

Ninety-four out of 108 SC from 9 patients were evaluable. Four primary tumors were in the oropharynx, 2 in the larynx, 2 in the hypopharynx, and one in the nasopharynx. The patients were between 31 and 87 years old (average 61.4 years). All patients but one were diagnosed with UICC stage III or IV HNC by clinical and radiologic evaluation. The cervical lymph nodes were involded in 8/9 cases, distant metastasis was found in one patient. All but one oropharyngeal carcinoma were p16 negative. Treatment after initial staging involved surgery with curative intent in 5 cases, adjuvant radiation or chemoradiation in 4 cases and primary Chemoradiation in 3 cases. SC were generated from each of the tumor explants (**Table 1**).

### 3.1 Gross slice quality

**Figure 2:**
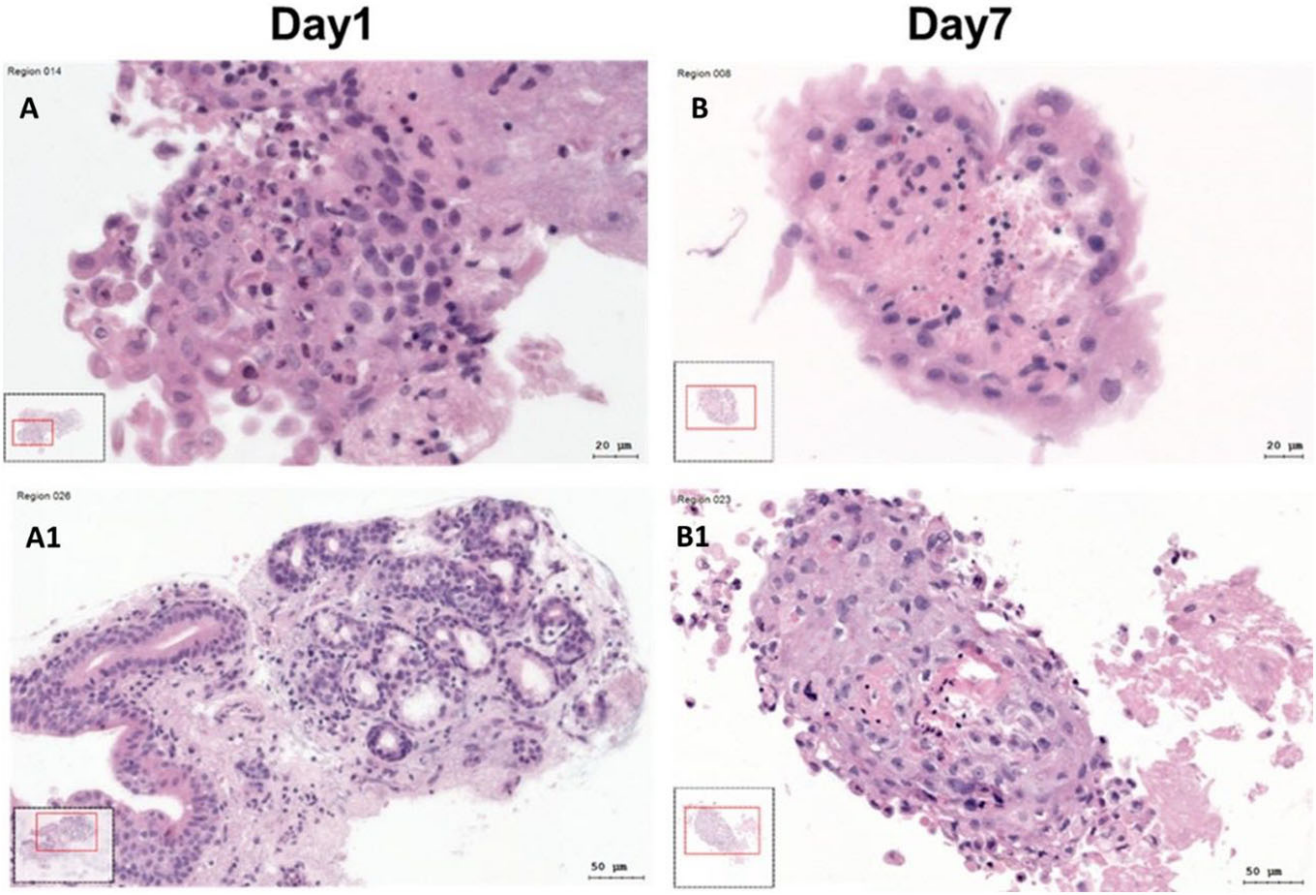
Hematoxylin–Eosin (HE) stained SC at day 1 and day 7. **(A, A1)** HE stained SC at day 1. **(B, B1)** HE stained SC at day 7.

Microscopic evaluation of gross SC quality in HE stainings revealed differences in quality and tissue composition depending on incubation time. After 24h, the tissue was mostly compact with almost nonnecrotic areas (**Figure 2**). After 7 days, the tissue structure and quality were still compact, but there were slightly more necrotic and dissembled areas visible (**Figure 2**).

Both in parametric and non-parametric tests, there was a significant correlation of HE quality score grade with SC total area. (Spearman´s rho=0.39; n=94; p<0.001). These tests also revealed a statistically significant correlation of HE quality scoring grades with the cell density (Spearman´s rho=0.6; n=94; p<0.001).

### 3.2 Effects of cultivation time, cultivation medium, and PRF on SC total area and cell density

#### 3.2.1 SC total area

**Figure 3:**
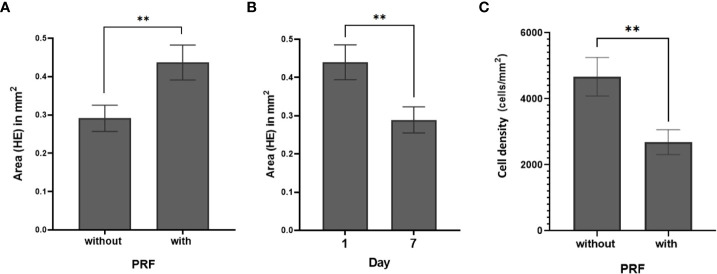
SC total area (mm2) (HE) **(A)** with and without PRF (p=0.009). **(B)** at day 1 and day 7 (p=0.007) **(C)** Cell density with and without PRF (p=0.003). (**significant at the 0.05 level; Bars: SEM).

Both cultivation time and addition of PRF had significant effects on the SC total area. Total area decreased from 0.44 ± 0.04 mm2 on day 1 (95% CI: 0.35 to 0.56) to 0.29 ± 0.03 mm2 on day 7 (95% CI: 0.22 to 0.36; p=0.007), (**Figure 3B**). SC total area was larger when cultivated with PRF (0.43 ± 0.05 mm2; 95% CI: 0.34 to 0.55; p=0.009) than without PRF (0.29 ± 0.03 mm2; 95% CI: 0.22 to 0.37; **Figure 3A**). This effect was independent of cultivation time. No significant effect of cultivation medium on SC total area and cell count was observed (p=0.205). Detailed patient specific data, in correspondence to **Figures 3A, B**, can be found in **Supplementary Table S1**.

#### 3.2.2 SC cell density

**Figure 4:**
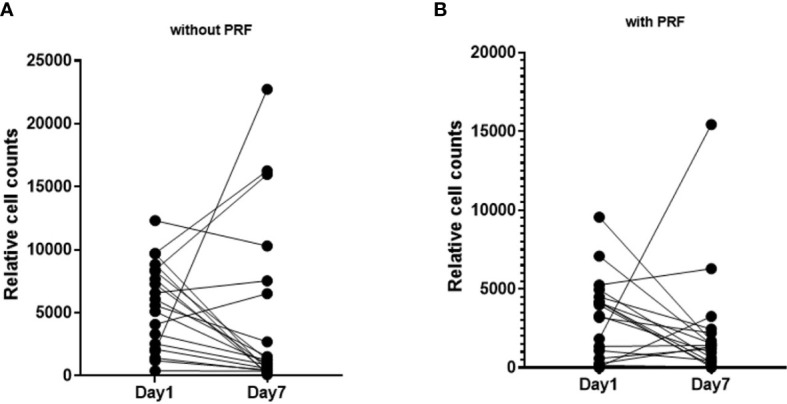
Pairwise visualization of slice cultures after 1 day and on day 7 without and with PRF. **(A)** Cell density of slice cultures without PRF on day 1 and day 7. **(B)** Cell density of slice cultures with PRF on day 1 and day 7.

Cell density was calculated by relating absolute cell count and SC total area. In all 94 slices, the mean cell density was 3544 ± 334 cells/mm2. PRF reduced cell density (p=0.003). Without PRF, 4669 ± 584 cells/mm2 (95% CI: 3654 to 5965) were counted in the slice cultures; whereas with PRF, 2690 ± 377 cells/mm2 (95% CI: 2045 to 3538) were observed (**Figure 3C**; **Supplementary Table S2**). Comparative visualization of SC after 1 and 7 days suggested increased cell counts in some of the PRF free, but barely in PRF containing SC (**Figures 4A, B**).

### 3.3 Effects of cultivation medium, cultivation time and PRF and on cell viability

**Figure 5:**
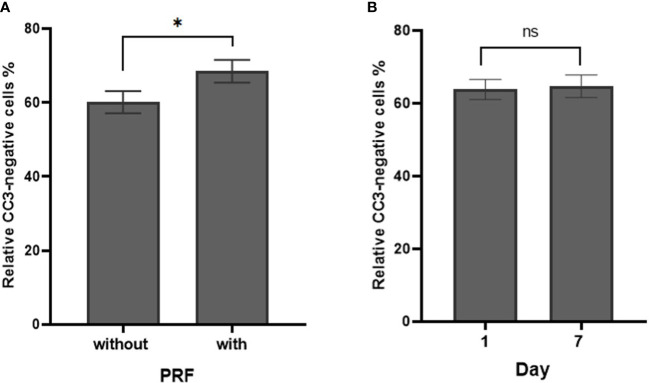
Cell density of CC3-negative stained cells in percentage in dependence of PRF and culture time. **(A)** Percentage of relative CC3-negative stained cells without and with PRF in percent (p=0.05). **(B)** Percentage of relative CC3-negative stained cells on day 1 and day 7 (p=0.834). (*significant at the 0.1 level, ns, not significant; Bars: SEM).

CC3 negative cells were considered viable and viability was calculated as the proportion of CC3 negative cells. Seventy-six SC were included in the viability analysis. Overall cell viability was 64.3 ± 2.1% (95% CI: 60.1 to 68.5). PRF increased cell viability (p=0.05). Cell viability was 60.1 ± 3% (95% CI: 54.2 to 66) without PRF vs. 68.48 ± 3.06% (95% CI: 62.5 to 75.5) with PRF (**Figure 5A**). There was no significant effect of cultivation media and cultivation time on cell viability (p=0.546 and p=0.834, respectively). Cell viability remained stable between 1 day (63.8 ± 2.8%; 95% CI: 58.2 to 69.5) and 7 days (64.7 ± 3.1%; 95% CI: 58.6 to 70.8; **Figure 5B**). Detailed patient specific data, in correspondence to **Figures 5A, B**, can be found in **Supplementary Table S3**.

### 3.4 Effects of cultivation time, cultivation medium, and PRF on different cell types

Effects of the three media, addition of PRF, and cultivation period on proportions of epithelial cells, mesenchymal cells and immune cells were also studied. There was a significant effect of the cultivation medium on leukocytes density (p=0.02). Highest leukocyte density were detected when SC were cultivated in combined medium (2686 ± 432 leukocytes/mm2; 95% CI: 1839 to 3534), compared to cultivation in Keratinocyte (1154 ± 367; 95% CI: 434 to 1873) and RPMI-1640 (1449 ± 445; 95% CI: 575 to 2322) media, respectively. Culture time and addition of PRF did not influence leukocyte density (p=0.61 and 0.498 respectively). No significant effect of cultivation medium, time, or PRF on tumor cells and fibroblast density were observed (p-values 0.105-0.835 and 0.485-0.901 for tumor cells and fibroblasts, respectively).

**Figure 6:**
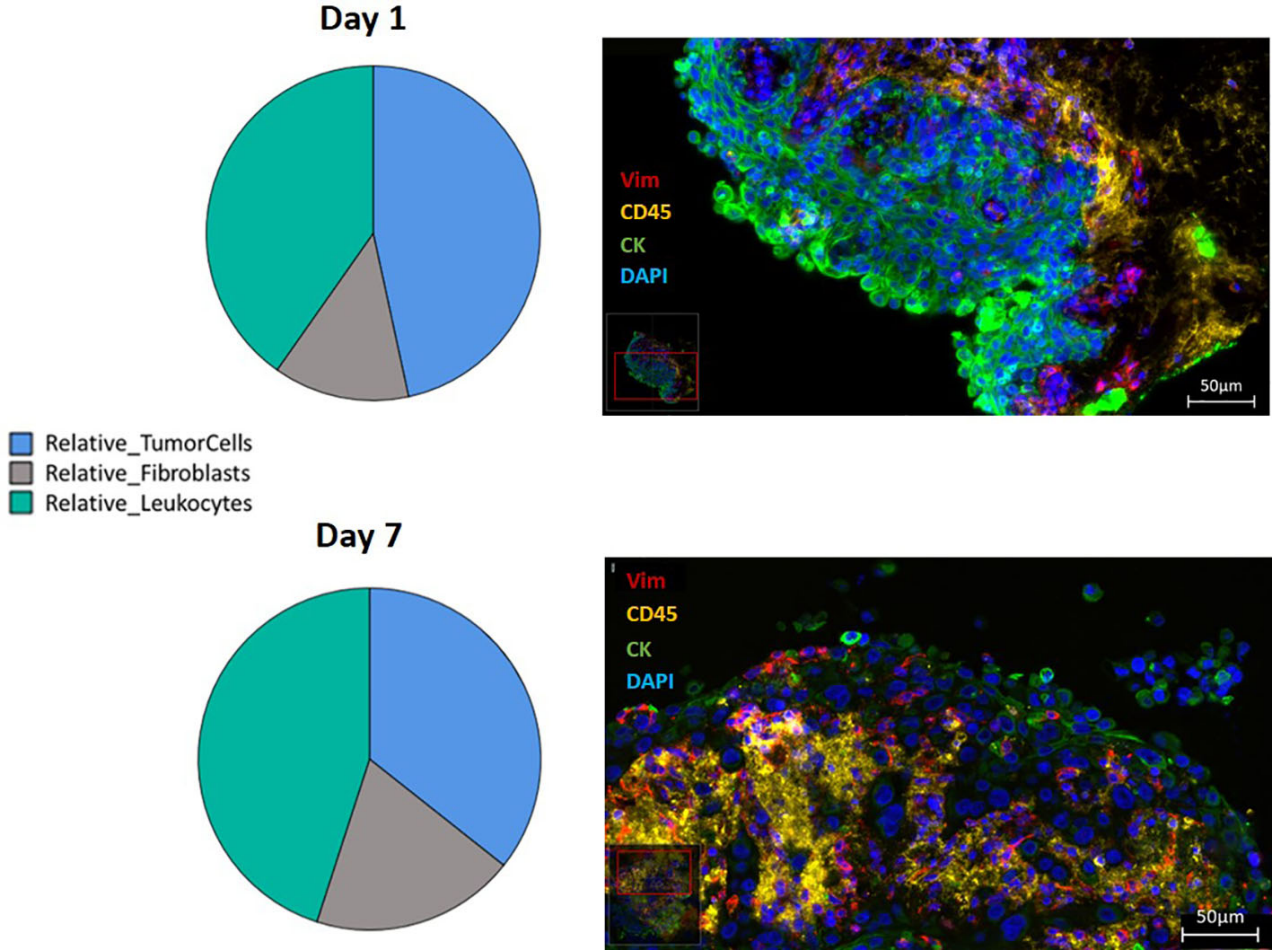
Relative distribution of the three different cell types, presented in a pie-diagram and IF triple staining using direct conjugated antibodies (CD45 conjugated with Alexa Fluor 594 presented in yellow, vimentin conjugated with eFluor 570 presented in red, pan-cytokeratin conjugated with Alexa Fluor 488 presented in green and counterstained with DAPI presented in blue) of SC from HNSCC at day 1 and day 7 (400 times magnification; Scale bars: 50µm).

Proportions of tumor cells, leukocytes, and fibroblasts were investigated in the context of cultivation time, medium, and addition of PRF. Culture time decreased the relative amount of tumor cells (p=0.046). After 1 day and 7 days, tumor cells presented 33% ± 3.02 (95% CI: 27.4 to 39.2) and 25% ± 3.19 (95% CI: 18.3 to 30.8) of all DAPI-positive cells. There was no significant effect of cultivation time, media, or PRF on the relative amounts of fibroblasts and leukocytes (**Figure 6**).

## 4 Discussion

Due to their preserved tissue architecture, native stroma, and heterogeneity, patient derived slice cultures may be suitable for testing patient specific treatments. HNC SC have been established previously as a platform to study HNC and its microenvironment and novel therapeutic approaches. Cytotoxic effects of high doses of Cisplatin, Docetxel and Cetuximab were studied by Gerlach et al. in HNC SC. According to their observations, they detected fragmentation of nuclei, pygnotic alterations and cellular polymorphisms as hallmarks of apoptosis. Recently, Runge, Mayr et al. reported on HNC SC as a platform to evaluate oncolytic virus action. In this study, it was possible to observe both tumor cell killing effects of the virus and lymphocytic tumor infiltration over the course of several days (29, 31). The goal of this study was to optimize HNC SC cultivation conditions and observe the effect of autologous PRF and different cultivation media on gross quality, viability, and composition of cell types in SC over a period of 7 days. This study was intended to set the ground works for stable culturing conditions over a course of several days, when effects of cytotoxic drugs and check point inhibitors are to be studied in close to real life *in vitro* conditions.

### 4.1 SC area and cell density, viability and proportions on day 1 and day 7

Gross slice quality, as estimated by HE stainings, remained stable for 7 days with intact structure, discernible cell types, and only a few necrotic areas. An earlier cultivation cut-off due to reduced SC quality, as described by Gerlach and coauthors, was not necessary (29). However, culturing time influenced SC total area and SC cell density. The initial cutting process may trigger inflammatory immune responses in SC, and modify cell growth and/or survival. While the TME can remain intact, SC total area may decrease over the course of 7 days, as observed in this and previous studies (29, 37, 40). However, viability and cell proportions, including the tumor cells as primary therapeutic targets, did not vary greatly between day 1 and day 7. As a result, future preclinical targeted therapeutic trials using HNC SC may be less biased by *in vitro* cell death or shifts in cell proportions. Still, according to our observations and similar earlier studies, SC quality is best preserved after 24-48 hours. Thus experiments need to be performed at early time points in the culturing period for optimal results (31). Finally, the impact of decreased SC size should be considered when calculating the effect of cytotoxic or immune modulating therapies in HNC SC.

### 4.2 SC area, cell viability, density and proportions with and without PRF

However, the addition of PRF was beneficial for preserving the SC total areas (p=0.023) and cell viability over several days. Similar effects have been observed in SC with fetal bovine serum (FBS) in other studies (41). FBS is a widely used cultivation medium rich in growth factors, but its use is not encouraged due to ethical concerns and the presence of highly variable amounts of xenogeneic components which may cause adverse immunological effects. Autologous growth factors, like those contained in PRF, may contribute to conservation of the original tumor size and cell viability by stimulating a biologically active “in situ” environment.

After 7 days and addition of PRF, an overall lower cell density was observed (**Figure 4**). Still, cell density increased in some SC, regardless of PRF. Standardization of growth factors and cell counts both in PRF and in HNC SC is hardly feasible, considering the means of material collection. As a result, cell counts may vary during longer cultivation periods.

According to our findings, PRF may have a stabilizing effect on cell proportions in HNC SC, even during longer cultivation periods. This finding contradicts observations of cell proliferation of osteoblasts and fibroblasts (42, 43). However, remodeling may occur after time frames longer than 7 days, which was beyond the scope of this study.

### 4.3 SC area and cell density, viability and proportions in different cultivation media

Cultivation media on the other hand had no such impact on the SC total area, cell density or viability. Keratinocyte SFM supposedly supports the growth of anchorage independent cancer stem cells and epithelial cells in cell cultures and spheroids. This medium contains all essential nutrients and supplements for culturing. Addition of FBS is not necessary, rendering cultivation conditions consistent throughout the whole experiment. RPMI on the other hand was supplemented with additional FBS. This medium is known to promote the growth of many different cell types, esp. fibroblasts and leukocytes in culture (44). In our study, Keratinocyte SFM, RPMI +FBS and the 1:1 mix of both did not promote any specific cell type in HNSCC SC. However, this may be different for primary cell culture. Robust cell viability regardless of cultivation media may be attributed to preservation of intercellular signaling and contacts as well as protective structures such as extracellular matrix in the tumor microenvironment of the HNSCC SC. Our results reveal that out of the three media, Keratinocyte SFM would be beneficial for SC culturing, due to its neutral effects and FBS-free conditions.

For future experiments, frequent SC movement during the culturing period should be considered. Kishan et al. observed facilitated nutrient exchange between the medium and SC and increased cell proliferation in floating SC installed in a shaker (45). These results may be applicable to tumors that are subject to passive nutrient distribution by constant muscle action, such as those in the head and neck region.

### 4.4 Strengths

HNC is characterized by heterogeneity in terms of patient and tissue characteristics, which can be fully represented in HNC SC. A cultivation period of up to 7 days allows for sufficient observation of treatment effects. Cultivation media seem to be interchangeable without generating major changes in the SC. A standard biopsy of > 4 mm³, taken during routine endoscopy under anesthesia, procures up to 12 slices with 300 µm. Out of these slices it was possible to generate 94 SC with good quality.

### 4.5 Limitations

The majority (94) of the planned 108 slices were applicable for cultivation. However, some slices were lost due to squashing or disintegration during initial cutting and staining or after cultivation for 7 days. Therefore, despite the already good quality slice cultures the application of the Compresstome^®^ vibrating microtome (Precisionary, MA, USA) yielded a high number of slices for further processing and statistically significant results (unpublished additional information). The consistency of tumor tissue was highly variable, and some samples were harder to cut than others. Consequently, an individual quality scoring had to be established to estimate SC quality, which was subject to observational bias and may have led to exclusion of further samples to achieve comparability. Additional input parameters, such as movement, longer incubation time, and additional media may be necessary to further optimize HNC SC conditions.

## 5 Conclusions

Despite high inter-individual variations in texture and cellular proportions, cultivation of HNSCC slice cultures up to 7 days with a useful number of viable cells is possible. As cultivation media did not impact SC quality, HNC SC may be highly versatile tools for studying physiology and drug effects in HNC. The addition of PRF provides an environment comparable to *in situ* conditions of the tumor and further stabilizes SC size and cell proportions. These results may aid in future experiments on patient-based therapy planning.

## Data availability statement

The raw data supporting the conclusions of this article will be made available by the authors, without undue reservation.

## Ethics statement

The studies involving human participants were reviewed and approved by Ethics committee of the Medical University of Innsbruck. The patients/participants provided their written informed consent to participate in this study.

## Author contributions

MG - methodology, investigation, data curation, writing- original draft, review and editing, visualization, project administration, formal analysis. AnR – methodology, investigation, writing- original draft, writing- review and editing, project administration, project administration, formal analysis. JD - methodology, investigation, data curation, resources, writing- editing, project administration. LC - investigation, data curation, formal analysis, writing- original draft. AvR - interpretation of data, formal analysis, writing-review and editing. MM - conceptualization, methodology, formal analysis, writing- review and editing. MP - methodology, investigation, resources, writing- review and editing. HR - Conceptualization, methodology, formal analysis, data curation, supervision, validation, writing-review and editing. VS - conzeptualization, material acquisition, writing- review and editing. All authors contributed to the article and approved the submitted version.
