# Supplementary material for: Optimizing culturing conditions in patient derived 3D primary slice cultures of head and neck cancer
Source: Front Oncol. 2023 Mar 30;13:1145817. doi: 10.3389/fonc.2023.1145817 (PMC10101142; doi:10.3389/fonc.2023.1145817)
Supplement: Supplementary file 1 [file DataSheet_1.docx]

## **Supplementary Material**

### Supplementary Table S1

Detailed list of the mean SC total area for all patients’ samples cultured for 1 day and 7 days with and without PRF, corresponding to Figure 3 A and B. When cultured with PRF, SC total area was generally larger both after 1 day and 7 days than after culturing without PRF. (day 1: 7 patients, day 7: 6 patients). However, in 8/9 patients, SC total area was reduced at day 7 compared to day 1 if the SC were cultured with PRF. These results indicate that the positive effect of PRF on the SC total area is time limited.

|  | | Day 1 | | Day 7 | |
| --- | --- | --- | --- | --- | --- |
|  |  | without PRF | with PRF | without PRF | with PRF |
|  |  | mean total area (mm^2^) | mean total area (mm^2^) | mean total area (mm^2^) | mean total area (mm^2^) |
| Patient | 1 | 0.30600 | 0.46787 | 0.27840 | 0.40747 |
|  | 2 | 0.16213 | 0.31615 | 0.18017 | 0.12993 |
|  | 3 | 0.04913 | 0.42270 | 0.21243 | 0.19687 |
|  | 4 | 0.41430 | 1.16427 | 0.57800 | 0.87310 |
|  | 5 | 0.30620 | 0.47580 | 0.05490 | 0.39255 |
|  | 6 | 0.98947 | 0.85527 | 0.26830 | 0.29880 |
|  | 7 | 0.30075 | 0.16542 | 0.28065 | 0.28085 |
|  | 8 | 0.14165 | 0.85540 | 0.21540 | 0.39103 |
|  | 9 | 0.34320 | 0.37659 | 0.36795 | 0.12660 |

### Supplementary Table S2

Detailed list of the mean cell density (cells/mm^2^) for all patients’ samples cultured with and without PRF, corresponding to Figure 3 C. A negative effect of PRF on cell density was found for 8 out of 9 patients.

|  | | without PRF | with PRF |
| --- | --- | --- | --- |
|  |  | Mean cell density (cells/mm^2^) | Mean cell density (cells/mm^2^) |
| Patient | 1 | 10983.80 | 4745.89 |
|  | 2 | 3265.85 | 1009.39 |
|  | 3 | 7171.46 | 584.61 |
|  | 4 | 1787.32 | 1414.27 |
|  | 5 | 1759.46 | 1505.85 |
|  | 6 | 3862.95 | 2940.81 |
|  | 7 | 1177.00 | 4836.62 |
|  | 8 | 5919.52 | 3972.44 |
|  | 9 | 7498.93 | 5035.75 |

### Supplementary Table S3

Detailed list of the mean percentage of viable cells for all patients’ samples after a culturing period of one day and seven days with and without PRF, corresponding to Figure 5 A and B. A positive effect of PRF on cell viability in the SC was observed in 6/8 patients after day 1 and in 4/9 patients after 7 days of culturing.

|  | | Day 1 | | Day 7 | |
| --- | --- | --- | --- | --- | --- |
|  |  | without PRF | with PRF | without PRF | with PRF |
|  |  | viable cells (%) | viable cells (%) | viable cells (%) | viable cells (%) |
| Patient | 1 | 43 | 73 | 49 | 42 |
|  | 2 | 58 | 52 | 41 | 72 |
|  | 3 | 63 | 84 | 63 | 75 |
|  | 4 | 58 | 47 | 47 | 54 |
|  | 5 | 59 | 87 | 64 | 85 |
|  | 6 | 64 | 79 | 69 | 88 |
|  | 7 | 51 | 60 | 91 | 69 |
|  | 8 | 64 | n.a. | 63 | 55 |
|  | 9 | 74 | 85 | 74 | 72 |
